# Ebola at a critical juncture: preparedness must outpace transmission

**DOI:** 10.2471/BLT.26.296555

**Published:** 2026-07-01

**Authors:** Herry Susanto, Mega Hasanul Huda, Minh Cuong Duong, Tri Nugraha Susilawati

**Affiliations:** a Department of Pediatric Nursing, Universitas Islam Sultan Agung, Semarang, Indonesia.; b Department of Pediatric Nursing, Universitas Indonesia, Jakarta, Indonesia.; c School of Population and Health, Faculty of Medicine and Health, University of New South Wales, Sydney, Australia.; d Department of Microbiology, Faculty of Medicine, Universitas Sebelas Maret, Jl. Ir. Sutami 36A Surakarta, Central Java, 57126, Indonesia.

The ongoing Ebola disease outbreak in the Democratic Republic of the Congo and Uganda is a critical test of whether, after the coronavirus disease 2019 pandemic, global health security has transitioned from reactive response to resilient preparedness. On 17 May 2026, the World Health Organization (WHO) declared Ebola disease caused by the Bundibugyo virus a Public Health Emergency of International Concern (PHEIC), citing confirmed cross-border transmission, suspected deaths, incomplete epidemiological linkages and probable underdetection.^1^ The United States Centers for Disease Control and Prevention and WHO subsequently mobilized support for surveillance, laboratory diagnostics, infection prevention and control and containment.^1^^,^^2^ However, the absence of approved Bundibugyo-specific vaccines or therapeutics underscores a persistent vulnerability in the global countermeasure landscape.^1^

Although Ebola outbreaks recur, their escalation follows well-established patterns. Delayed detection, fragile surveillance systems, suboptimal infection prevention and control, population mobility, insecurity and community distrust repeatedly converge to amplify transmission. During the 2018–2019 outbreaks in North Kivu and Ituri, insecurity and mistrust hindered case identification, contact tracing, care-seeking and vaccination uptake.^3^ Historical analyses across outbreaks in the Democratic Republic of the Congo further demonstrate that nosocomial amplification (that is, when an infectious disease outbreak spreads within a health-care setting) is often a defining early feature, reflecting gaps in infection control capacity.^4^ Evidence from the Democratic Republic of the Congo and Sierra Leone reinforces the notion that health-care settings can inadvertently drive transmission when infection prevention and control adherence is inconsistent and health workers are insufficiently protected.^5^^,^^6^

The current PHEIC should therefore be interpreted as an emergency alert as well as a signal of structural insufficiency. Preparedness remains episodic in the affected areas, that is, activated in crisis and deprioritized thereafter, rather than embedded as a sustained, system-wide capability.^1^ Experiences from Uganda’s 2018–2019 preparedness and readiness programme demonstrate that proactive investments such as cross-border risk mapping, strengthened points of entry and exit in the affected areas through screening, containment and treatment, district-level readiness assessments and community engagement platforms can enhance anticipatory capacity, particularly in low-resource areas.^7^^,^^8^ Crucially, such preparedness and readiness systems are most effective when maintained, financed and routinely exercised between outbreaks, rather than improvised in response to them.

We suggest that a shift from reactive to resilient preparedness requires prioritization of four interlocking actions. First, governments in the Democratic Republic of the Congo, Uganda and neighbouring countries should strengthen joint cross-border surveillance, interoperable data systems and real-time information sharing, alongside decentralized laboratory capacity to reduce delays. Second, health-care facilities must be reinforced through sustained infection prevention and control investments, including triage systems, isolation capacity, personal protective equipment, safe patient referral, workforce training and supportive supervision. Third, community engagement must evolve from one-directional risk communication to genuine co-production involving local leaders, survivors and community health workers in both preparedness and response. Recent evidence from Uganda highlights that engagement efforts are often weakened by misinformation, limited feedback mechanisms and insufficient inclusion of local actors.^9^ Fourth, the absence of approved Bundibugyo-specific treatment and vaccines should accelerate ethically governed clinical research while reaffirming that high-quality supportive care, safe and dignified burials, and robust infection prevention and control remain the foundation of outbreak control.^1^^,^^10^

Recent analyses of the 16th Ebola outbreak that was declared on 4 September 2025 in the Democratic Republic of the Congo emphasize the importance of integrating genomic surveillance, community perspectives and lessons learnt into a coherent preparedness roadmap.^11^ This outbreak shows that despite accumulated experience and advances such as rapid genomic sequencing, the absence of an integrated preparedness roadmap led to persistent gaps in detection, coordination and community engagement. Without a pre-established, operational roadmap linking these elements, responses will remain reactive rather than preventive. On the other hand, pandemic-prone threats such as Ebola require preparedness architectures that are continuous, adaptive and embedded within resilient primary health-care systems, laboratory networks, emergency operations and trusted community structures. National governments through health ministries, regional partners and the global health community must be involved in promoting the shift from reactive response to resilient pandemic preparedness. Without this shift, global health security will remain in a reactive state that responds to outbreaks rather than prevents their amplification.
